# A Research Domain Criteria Approach to Gambling Disorder and Behavioral Addictions: Decision-Making, Response Inhibition, and the Role of Cannabidiol

**DOI:** 10.3389/fpsyt.2021.634418

**Published:** 2021-09-17

**Authors:** Stefano Pallanti, Anna Marras, Nikolaos Makris

**Affiliations:** ^1^Institute of Neurosciences, Florence, Italy; ^2^Albert Einstein College of Medicine and Montefiore Medical Center, New York, NY, United States; ^3^Department of Neurosciences, Psychology, Drug Research and Child Health (NEUROFARBA), University of Florence, Florence, Italy; ^4^Departments of Psychiatry and Neurology, Center for Morphometric Analysis, Massachusetts General Hospital and Harvard Medical School, Boston, MA, United States

**Keywords:** gambling disorder, behavioral addictions, endocannabinoids, Research Domain Criteria, cannabidiol

## Abstract

Gambling Disorder (GD) has been recently re-classified in the DSM-5 under the “substance-related and addictive disorders,” in light of its genetic, endophenotypic, and phenotypic resemblances to substance dependence. Diminished control is a core defining concept of psychoactive substance dependence or addiction and has given rise to the concept of “behavioral” addictions, which are syndromes analogous to substance addiction, but with a behavioral focus other than ingestion of a psychoactive substance. The main symptom clusters are represented by loss of control, craving/withdrawal, and neglect of other areas of life, whereas in a Research Domain Criteria (RDoC) perspective, GD patients exhibit deficits in the domain of “Positive valence systems,” particularly in the “Approach motivation” and “Reward learning” constructs, as well as in the “Cognitive systems,” primarily in the “Cognitive control” construct. In the Addictions Neuroclinical Assessment (ANA), three relevant domains for addictions emerge: “Incentive salience,” “Negative Emotionality,” and “Executive Function.” The endocannabinoid system (ECS) may largely modulate these circuits, presenting a promising pharmaceutical avenue for treating addictions. Up to now, research on cannabidiol has shown some efficacy in Attention Deficit/Hyperactivity Disorder (ADHD), whereas in behavioral addictions its role has not been fully elucidated, as well as its precise action on RDoC domains. Herein, we review available evidence on RDoC domains affected in GD and behavioral addictions and summarize insights on the use of cannabidiol in those disorders and its potential mechanisms of action on reward, decisional, and sensorimotor processes.

## Introduction

Behavioral addictions refer to syndromes analogous to substance addiction, but with a behavioral focus other than ingestion of a psychoactive substance (1) and Gambling Disorder (GD) is often recognized as the prototypical behavioral addiction (2). The essential feature of behavioral addictions is the failure to resist an impulse, drive, or temptation to perform an act that is harmful to the person or to others. Each behavioral addiction is characterized by a recurrent pattern of behavior that has this essential feature within a specific domain. The repetitive engagement in these behaviors ultimately interferes with functioning in other domains. In this respect, the behavioral addictions resemble substance use disorders (1).

The diagnostic criteria for gambling disorder overlap largely with those for the substance use disorders: the main symptom clusters are represented by loss of control, craving/withdrawal, and neglect of other areas of life (3): there are commonalities between substance use disorders (SUDs), including the use of stimulants, alcohol, nicotine—and behavioral addictions including gambling, internet use, shopping, and eating, in terms of elements of automatized, dysregulated cognitions, and behaviors (4).

The inclusion of Gambling Disorder (GD) in the addictive disorder chapter of DSM-5 is motivated by the recognition of its genetic, endophenotypic, and phenotypic resemblances to substance dependence: both disorders show similar comorbidity patterns (5), genetic vulnerabilities, and responses to specific pharmacologic treatments (6).

The hallmark components of the disorder have been proposed to be (a) continued engagement in a behavior despite adverse consequences, (b) diminished self-control over engagement in the behavior, (c) compulsive engagement in the behavior, and (d) an appetitive urge or craving state prior to engaging in the behavior (7, 8).

Recently, a framework for an Addictions Neuroclinical Assessment (ANA) has been proposed (9). Three main neurofunctional domains, executive function, incentive salience, and negative emotionality, should be assessed in patients with addictions, including behavioral addictions (“process” addictions as defined by the American Society of Addiction Medicine, e.g., gambling) and in individuals at risk, for purposes of better understanding the heterogeneity of AD and eventually to improve the nosology.

The endocannabinoid system (ECS) has been shown to influence the acquisition and maintenance of drug-seeking behaviors, through its role in reward and brain plasticity. Cannabinoid receptors have been studied in addiction-related processes, with special attention paid to cannabinoid type 1 (CB1) receptors (CB1R). Other ionotropic cannabinoid receptors are also linked to neurophysiological functions in the ECS, such as transient receptor potential receptors, including transient receptor vanilloid potential 1 (TRVP1), which binds the endogenous cannabinoid anandamide (AEA) (10). Up to now, available evidence on the role of the ECS in GD and other behavioral addictions is still scarce and thus require a broadening of studies and a review of current results, in order to optimize treatment for those conditions and to consider the employ of cannabidiol and related compounds. In this review, we will briefly summarize the conceptualization of GD and behavioral addictions in a Research Domain Criteria (RDoC) framework, also considering the relevant neurocircuitry as candidate target for cannabidiol treatment and available evidence on the role of ECS and its dysregulations in those conditions.

## Gambling Disorder, Behavioral Addictions, and the Research Domain Criteria

Gambling Disorder is characterized by a persistent, recurrent pattern of gambling that is associated with substantial distress or impairment. It is currently classified within the addictive disorder chapter of DSM-5 and it is characterized by a maladaptive pattern of gambling behavior that persists despite negative consequences in major areas of life functioning. GD is highly comorbid with other psychiatric disorders. The strongest evidence relates GD to substance use disorders: pathological gamblers have an increased risk of having a diagnosis of alcohol misuse in lifetime and an increased risk of having a substance use disorder (11). The National Institute of Mental Health (NIMH) has recently launched the Research Domain Criteria (RDoC) project to overcome the limitations of current classification systems and to develop a framework for research on mental disorders that includes multiple dimensions (12): behavior, thought patterns, neurobiological measures, and genetics, with a strong focus on neurocircuitries. The RDoC aims at facilitating the incorporation of behavioral neuroscience in the study of psychopathology and at identifying reliable and valid psychological and biological mechanisms and their disruptions, with an eventual goal of understanding how abnormalities in these mechanisms drive psychiatric symptoms (13). RDoC's strong focus on neural circuits is evident from the assumption that mental illnesses are conceptualized as brain disorders of brain circuits. Moreover, the RDoC assumes that dysfunctions in neural circuits can/will be identified by tools of neuroscience (12). Importantly, in the RDoC approach, the behavioral and genetic phenotypes are bridged and integrated through specific brain circuitries, which embody the level of systems biology (14–17). Recently, the RDoC matrix has been extended to include a sixth domain referred as “Sensorimotor Systems” which “*are primarily responsible for the control and execution of motor behaviors, and their refinement during learning and development*” (18). The belonging constructs seem to be related mainly to stereotypic behaviors and/or tics.

**Figure 1:**
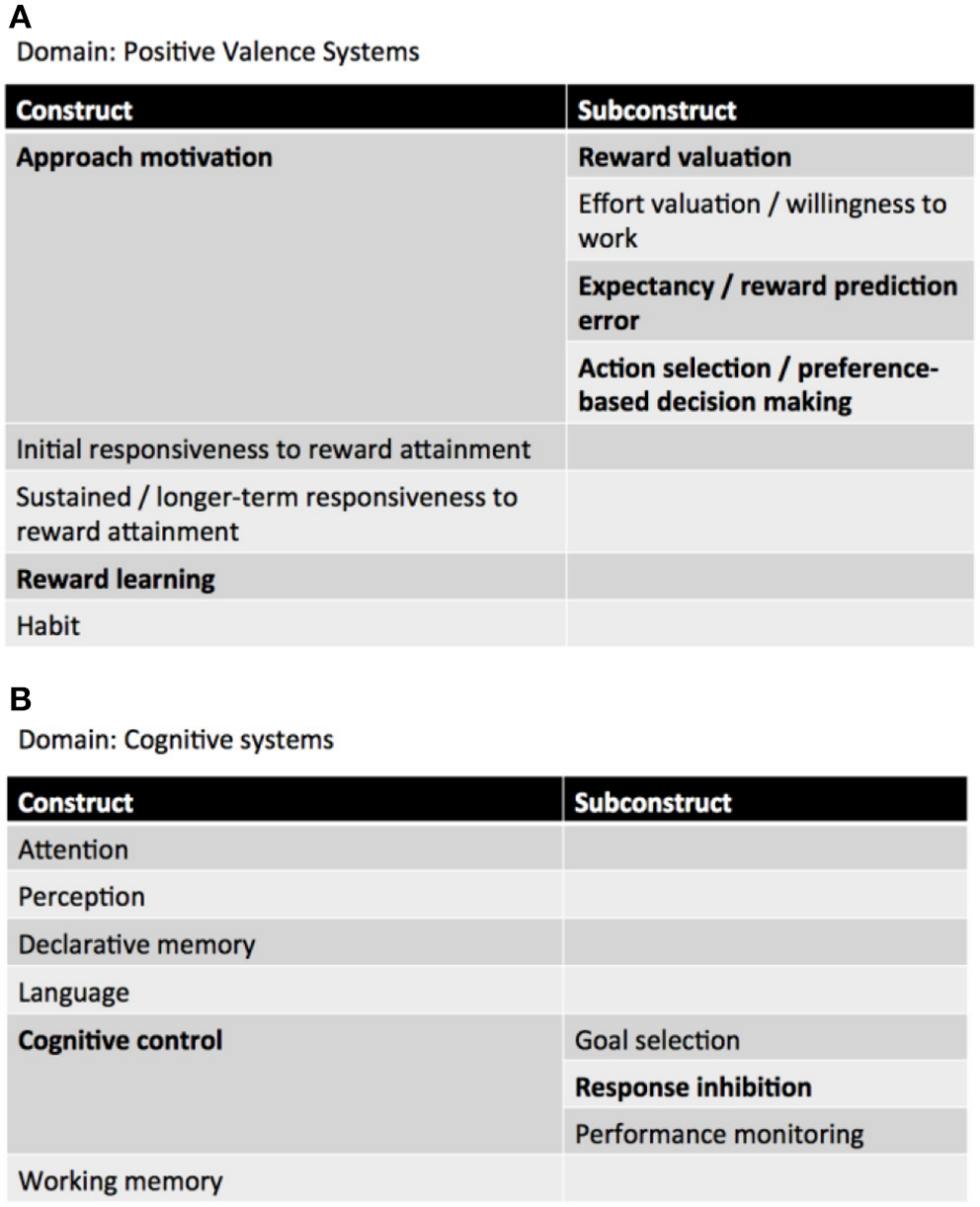
RDoC domains involved in GD. **(A)** Positive valence systems, **(B)** cognitive systems (adapted from: NIMH RDoC Matrix https://www.nimh.nih.gov/research-priorities/rdoc/constructs/rdoc-matrix.shtml).

Neurocircuitries are phenotypic targets of great potential for endophenotypic/biomarker discovery in current neuroimaging clinical research (19). In a RDoC perspective, patients with behavioral addictions—and GD—exhibit impairments in the domain of “Positive valence systems,” particularly in the “Approach motivation” and “Reward learning” constructs, as well as in the “Cognitive systems,” more specifically in the “Cognitive control” construct. Patients with Attention Deficit/Hyperactivity Disorder (ADHD) seem to display, as well, impairments in the domains of “Positive Valence Systems” (Reward anticipation, receipt, and delay) and “Cognitive systems” (Working memory) (20) (Figure 1).

Positive valence systems are primarily responsible for responses to motivational situations such as reward seeking, consummatory behavior, and reward/habit learning (18). The construct of Approach motivation involves “*mechanisms/processes that regulate the direction and maintenance of approach behavior influenced by pre-existing tendencies, learning, memory, stimulus characteristics, and deprivation states*” (ibidem). Particularly relevant to GD is the subconstruct Reward valuation, which consists of “*processes by which the probability and benefits of a prospective outcome are computed and calibrated by reference to external information, social context (e.g., group input, counterfactual comparisons), and/or prior experience. This calibration is influenced by pre-existing biases, learning, memory, stimulus characteristics, and deprivation states. Reward valuation may involve the assignment of incentive salience to stimuli*” (ibidem).

Cognitive systems are responsible for various cognitive processes. Specifically, cognitive control “*modulates the operation of other cognitive and emotional systems, in the service of goal-directed behavior, when prepotent modes of responding are not adequate to meet the demands of the current context. Additionally, control processes are engaged in the case of novel contexts, where appropriate responses need to be selected from among competing alternatives*” (21).

A complementary initiative to the RDoC is the Addictions Neuroclinical Assessment (ANA) (9), that incorporates key functional domains derived from the neurocircuitry of addiction. In this one, three domains (executive function, incentive salience, and negative emotionality) tied to different phases in the cycle of addiction, form the core functional elements of addictive disorders. The common point between RDoC and ANA is the consideration of neuroscience domains and the identification of meaningful subtypes of disorders.

## Gambling Disorder Domains: Behavioral Tasks and Neurocircuitry

### Positive Valence Systems

#### Approach Motivation: Preference-Based Decision-Making

In a RDoC perspective, these processes involve an evaluation of costs/benefits and occur in the context of multiple potential choices being available for decision-making (18).

Changes in reward based decision-making and increases in impulsivity are hallmark features of addiction (22) that has been scarcely studied satisfactorily in GD. Risky decision-making is a core feature of GD: gamblers have a high tolerance toward risk (23, 24) and a bias to select short-term over long-term rewards is integral to the syndrome (25). This bias has been operationalized with the employ of a behavioral measure called delay discounting task [DDT; (26)], in which participants choose between pairs of options that yield small, immediate vs. large, delayed rewards. Subjects with substance abuse and behavioral addictions show a tendency to choose small and immediate rewards rather than large and delayed rewards. The Iowa Gambling Task (IGT) (27) has also been employed as a measure of decision-making, since it is considered as the most widely used and ecologically valid measure of decision making in this clinical population. In the IGT, players are given four decks of cards and an endowment of fake money (e.g., $2,000) and are instructed to select cards one at a time and try to lose the least amount of money and win the most. GD subjects have shown to perform worse on the IGT and to make more high-risk choices compared to controls, precisely after experiencing wins and losses (28). During high-risk gambling decisions, fMRI has shown that, during the IGT task, GD subjects exhibit relatively increased frontal lobe and basal ganglia activation, particularly involving the orbitofrontal cortex (OFC), caudate and amygdala. Increased activation of regions encompassing the extended reward pathway in GD subjects (GDs) during high risk choices suggests that the persistence of GD may be due to the increased salience of immediate and greater potential monetary rewards relative to lower monetary rewards or potential future losses (ibidem). There is also considerable evidence that GDs discount delayed rewards steeper than healthy controls (29). Neuroimaging research has shown that GD is associated with a shift in the interplay between a prefrontal-parietal control network and a brain network involved in immediate reward consumption (30), and a generally hypoactive reward system (31).

A differential activation of distinguishable neural systems between immediate and delayed choices has been highlighted, with the former driven by the limbic system (including the ventral striatum, medial orbitofrontal cortex (MOFC), medial prefrontal cortex (MPFC), posterior cingulate cortex (PCC), and left posterior hippocampus) and the latter by the lateral prefrontal cortex and associated structures [including the right and left intraparietal cortex (RPar, LPar), right dorsolateral prefrontal cortex (DLPFC), right ventrolateral prefrontal cortex (VLPFC), and right lateral orbitofrontal cortex (LOFC)] (32).

More specifically, there is evidence that the right hemisphere plays an important role in inhibiting impulsive behavior and that the right DLPFC holds a certain role in the process of general decision-making (33). Although the pathophysiology of GD is not well-understood, studies have shown altered brain activity in prefrontal regions (primarily the DLPFC) of GD patients in response to gambling stimuli. Recently, a hypersensitivity to extreme gain–loss ratios of dorsal cortico-striatal network involved in action–outcome contingencies has been shown in gamblers (34).

#### Reward Learning

The similarity between GD and substance abuse has been repeatedly hypothesized on the basis of large overlaps between addictive manifestations of both disorders. Recently, an interesting contribution to a broader understanding of the neurocognitive features of GD, hypothesized a loss of willpower to resist gambling, deriving from a pathological usurpation of mechanisms of learning that under normal circumstances serve to shape survival behaviors related to the pursuit of rewards and the cues that predict them (35). This mechanism has been shown to be related with reward-based cognitive inflexibility, presumably resulting from an aberrant reward-based learning and observed as some kind of continuous gambling even in the face of increasing losses (36).

On a neurobiological perspective, reward-based cognitive inflexibility, has been associated with the orbitofrontal cortex (OFC) (37), the ventral prefrontal cortex (vPFC) (38), the ventrolateral prefrontal cortex (vl-PFC) (39) and is facilitated by dopaminergic activity in the ventral regions of the striatum (37, 38).

### Cognitive Control

#### Response Inhibition

Response inhibition refers to the ability to suppress behaviors that are inappropriate, unsafe, or no longer required (40). Recent findings suggest that the ability to suppress automatic responses could be critical to gambling addictive behavior (35). Whereas the increased sensitization toward gambling-related cues appears to be related to a hyperactivity of impulsive processes that may explain gamblers' motivation to seek out relevant reward (35), the unsuccessful efforts to reduce or stop gambling despite negative outcomes (19, 41–43) are thought to depend on a dysregulation of the so-called “reflective system,” and specifically, a faulty inhibitory control, responsible for inadequate efforts to control (or cut back or stop) gambling (ibidem).

Inhibitory control has been usually assessed with behavioral measures such as the Stop Signal Task (SST) (44), in which subjects perform a choice reaction task, and, on a random selection of the trials, an auditory stop signal instructs subjects to withhold their response, or Go/No-Go tasks, which require people to make manual responses to rapidly presented visual or auditory cues (i.e., “Go” stimuli), but to withhold responses in the presence of a different cue (“No-Go” stimuli) (45).

Deficits in behavioral and cognitive control constitute a symptom dimension associated with diminished response inhibition in experimental tasks. Impaired response inhibition performance (i.e., prolonged latency of motor response inhibition) has been previously highlighted in pathological gambling by using the stop-signal task and the go/no-go paradigm [for a review, see (35)] and recent contributions highlight the correlation between deficits in response inhibition and gambling severity (46, 47).

Recent neuroimaging research suggests that response inhibition may depend on a fronto-basal-ganglia circuit, including the inferior frontal gyrus (IFG), the pre-supplementary motor area (pre-SMA) and the subthalamic nucleus (STN) and striatum (48). Both right IFG and pre-SMA activation appear to be associated with successful stop trials. However, whereas right IFG contributes to response inhibition and not to monitoring performance or adjusting behavior, the pre-SMA seem to be involved in monitoring or resolving the conflict between the opposing task demands in the stop-signal paradigm. Also, fMRI studies showed inhibition-related activation in basal ganglia, including the STN and striatum and lesions to the basal ganglia impaired stop performance for both humans and rodents (ibidem).

The concept of “loss of control” (LOC) reflects a psychopathology construct that is uniquely associated with distress and impairment and that, in eating disorders, is defined as a subjective experience of loss of control irrespective of the actual amount of food consumed (49). LOC has been extensively investigated in other consummatory behaviors, such as eating behaviors, where LOC frequently occurs in response to negative emotions in youth and then in adults, is associated with emotional disregulation (ibidem).

The construct of LOC is also closely related to the concept of “perceived control,” since even with the absence of objective control, having the perception of control is sufficient to increase arousal and mobilize action; whereas perceiving the lack or loss of control leads to helplessness despite the presence of objective control (50). On the other hand, a crucial role in the loss of control is the motor component, which reflects the construct of inhibitory control and is associated with decreased functionality of the prefrontal cortex, which involves an impaired ability to control behaviors (51–53). Disruption of the PFC in addiction underlies not only compulsive drug taking but also accounts for the disadvantageous behaviors that are associated with addiction and the erosion of free will (53). The role of inhibitory control in relation to the development and maintenance of loss of control over behavior is still to be fully elucidated, as well as the role of automatic processes as potential mediating factors (54). Herein, we focused on the symptom cluster “loss of control” (i.e., unsuccessful efforts to control, cut back, or stop gambling), which appears to be mainly related to impaired reward-related decision-making and deficits in executive functions. What is crucial to understand in regard to behavioral addictions is which component of LOC is predominant and in which phase of addiction and, more important, if there is any specificity for the affective or motor dimension to certain behavioral addictions. This could help in dissociating the neurocircuitry for those disorders, focusing more on reward-related-basal ganglia loops or on the prefrontal-orbitofrontal networks.

## The Endocannabinoid System

The endocannabinoid system (ECS) is a widespread neuromodulatory system that plays important roles in central nervous system (CNS) development, synaptic plasticity, and the response to endogenous and environmental insults. The ECS is comprised of cannabinoid receptors, endogenous cannabinoids (endocannabinoids), and the enzymes responsible for the synthesis and degradation of the endocannabinoids. perturbations of the ECS are involved in several psychiatric disorders, including schizophrenia (55). The most relevant receptors are CB1R and CB2R: while CB1R are abundant in the central nervous system (CNS), particularly in cortex, basal ganglia, hippocampus, and cerebellum, CB2R are expressed at much lower levels in the CNS compared to CB1R, and are primarily present in microglia and vascular elements (ibidem). The compound Δ9-tetrahydrocannabinol (THC) is the main psychoactive compound of *Cannabis sativa* L., whereas cannabidiol is one of the most abundant phytocannabinoids isolated from *Cannabis sativa L*. (up to 40% of the extract). In contrast with THC, cannabidiol does not exhibit psychomimetic activities. Several studies show CBD to have anti-inflammatory, anticonvulsant, antioxidant, antiemetic, anxiolytic, and antipsychotic properties; thus, it may serve as potential drug for the treatment of neuro-inflammation, epilepsy, oxidative injury, vomiting and nausea, and anxiety and schizophrenia, respectively (56).

### Endocannabinoid Signaling and Reward

Both exogenous AEA and 2-arachidonoyl glycerol (2-AG) increase extracellular dopamine levels in the nucleus accumbens in a CB1R-dependent manner and the ECS exerts a strong influence on the fine-tuning of midbrain dopamine cell activity. Through these and other interactions the ECS has a prominent influence on the hedonic effects of natural rewards such as food, sexual activity, and social interaction. This is mediated in part through a direct CB1R modulation of the mesolimbic dopamine response to natural reward and through the interactions between the ECS and other signaling systems (endogenous opioids, hypothalamic signaling molecules, etc.) (57). Although enhancement of endocannabinoids (EC) levels does not produce rewarding effects *per se*, EC signaling at cannabinoid receptors participates in the mediation and modulation of both natural and drug-induced reward. Brain EC content is modulated by most drugs of abuse and natural rewards and a robust CB1R influence on the motivation to consume distinct classes of abused drugs and the association of CNR1 gene polymorphisms with aberrant reward processing and addictive behaviors strongly implicates CB1Rs in the etiology of addiction (ibidem). Also several studies have suggested an association between acute or chronic use of exogenous cannabinoids (THC) and executive impairments, and a relevant modulation of the endocannabinoid system on prefrontal-dependent cognitive functioning and executive functioning has been highlighted (58).

## The Endocannabinoid System and the RDoC

**Figure 2:**
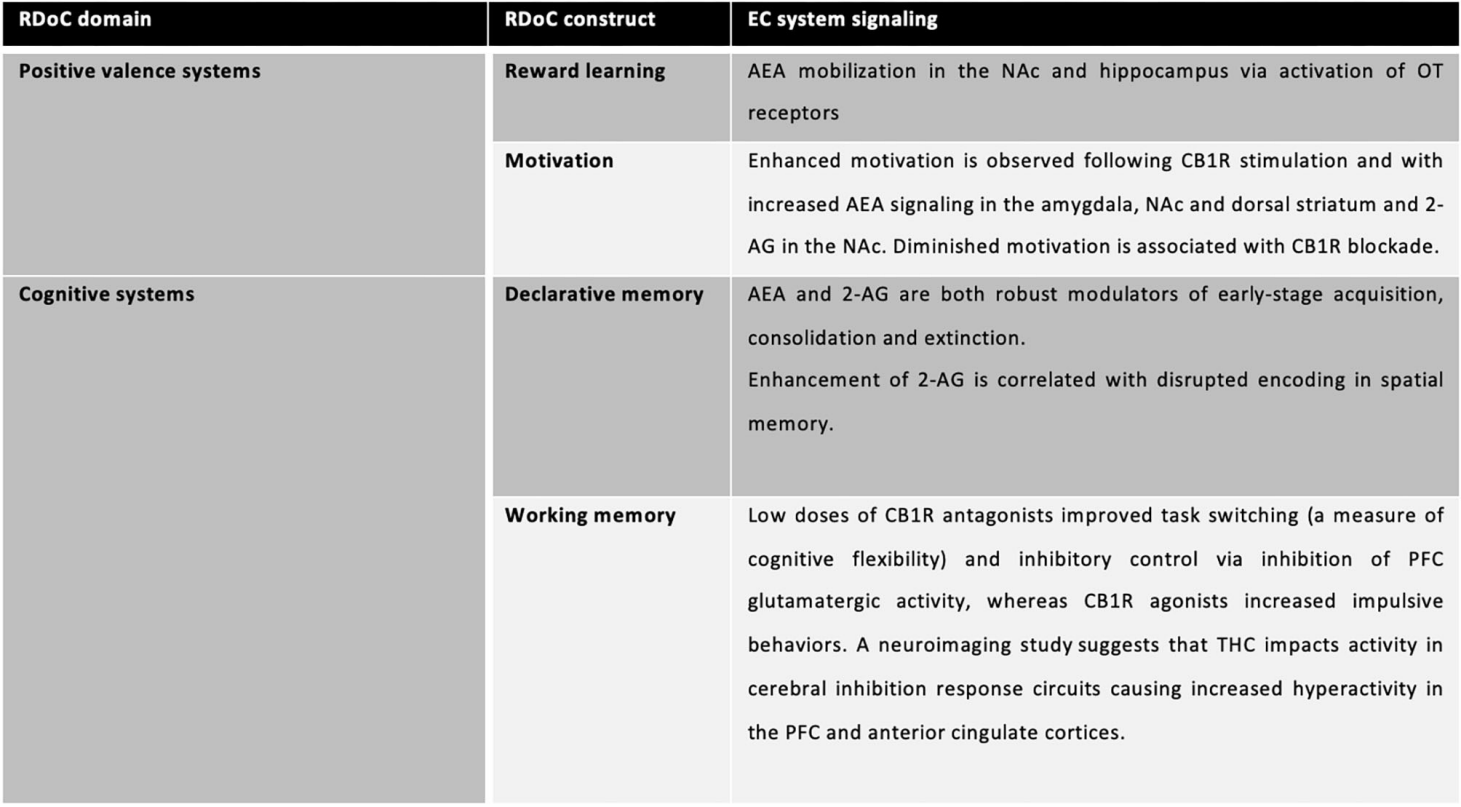
EC systems signaling involved in specific RDoC domains and constructs.

Endocannabinoid functioning has been recently studied in a RDoC perspective (59): its role in Positive Valence Systems and Cognitive Systems has been highlighted. Specifically, *reward attainment* is one of the only RDoC constructs to explicitly detail endocannabinoids as candidate modulators of reward learning, valuation, and processing (ibidem). In regard to Cognitive systems and particularly, *declarative memory*, stimulation of cannabinoid receptors in hippocampal circuits diminishes glutamate release to below-threshold levels, inhibiting long-term potentiation necessary for encoding and abundance of evidence demonstrates transient, dose-dependent Δ9-tetrahydrocannabinol (THC)-induced memory impairments (with a tolerance effect in heavy users) and the contrasting absence of memory deficits following CBD administration. THC exposure in humans negatively impacts working memory *via* CB1R activation and inhibition of AEA reuptake. Correspondingly, rodent models with upregulated CB1R expression in the PFC, as well as CB1R knockout mice, demonstrate changes in cognitive flexibility. Low doses of CB1R antagonists improved task switching (a measure of cognitive flexibility) and inhibitory control *via* inhibition of PFC glutamatergic activity, whereas CB1R agonists increased impulsive behaviors. A neuroimaging study suggests that THC impacts activity in cerebral inhibition response circuits causing increased hyperactivity in the PFC and anterior cingulate cortices. Acute administration of THC reduces response inhibition (that is, increases behavioral impulsivity) and causes hyperactivity at dopaminergic synapses in the PFC (59) (Figure 2).

THC also induces impairments in decision-making, which are thought to be the result of cannabinoid CB1R activation (60). In rat model of IGT (rat gambling task—rGT), blockade of the CB1R produced a trend improvement in decision making in animals who preferred the advantageous task options, yet left choice unaffected in risk-prone rats. Neither CB1R agonist had strong effects on decision-making, but a high dose THC decreased premature responses (ibidem). These results show that acute modulation of CB1R has modest effects on choice and instead may play a substantive role in regulating impulsive responding. Animal models also shown that activation of the cannabinoid system in the nucleus accumbens (NAc) is capable to impair effort-based decision-making: rats trained in a T-maze cost-benefit decision making task were led to be less willing to invest the physical effort to gain large reward after administration of cannabinoid system agonist (61). The anterior cingulate cortex (ACC) and the orbitofrontal cortex (OFC) are also involved in decision-making and murine models employing cost-benefit T-maze decision-making task showed that CB1R activation in the ACC impaired decision making such that rats were less willing to invest physical effort to gain high reward. Similarly, CB1R activation in the OFC induced impulsive pattern of choice such that rats preferred small immediate rewards to large delayed rewards (62).

## Cannabidiol and Response Inhibition

Response inhibition, as mentioned before, refers to the ability to suppress behaviors that are inappropriate, unsafe, or no longer required (40). Whereas THC impairs performance on motor and response inhibition tasks, cannabidiol (CBD) does not impair motor or cognitive performance (63). The Go/No-Go task is a classical response inhibition paradigm that requires participants either to execute or inhibit a motor response and recent contributions have examined the differential effects of Δ-9-THC and CBD on regional brain activation during response inhibition tasks. In regard to the specific behavioral response, neither THC nor CBD had a significant effect on task performance, save for an effect on the frequency of left/right errors (ibidem). A previous study (64) investigated the acute effects of THC on four behavioral measures of impulsivity (including a Go/No-Go task) in recreational marijuana users. THC impaired performance on a Stop task but did not have a significant effect on Go/No-Go performance, suggesting that THC may increase certain forms of impulsive behavior more than others. However, it is suggested that THC attenuates the engagement of brain regions that mediate response inhibition and that CBD modulates function in regions not usually implicated in response inhibition.

Another study investigated the differential effects of Δ-9-THC and CBD on regional brain activation during a set of four tasks that engaged cognitive processes known to be affected by cannabis use: verbal memory, response inhibition, sensory processing, and emotional processing (65). Specifically, response inhibition was measured with the Go/No-Go task and opposite effects of THC and CBD were observed in the parahippocampal gyrus during response inhibition. Although the parahippocampal areas are not part of the response inhibition network, opposite effects of Δ-9-THC and CBD in the parahippocampal areas during the response inhibition task is consistent with the high density of CB1R in these regions (ibidem).

Animal models have been employed to study the role of the ECS in response inhibition: CB1R agonists and antagonists were tested in rats during a stop-signal paradigm (the main task employed to test response inhibition). Results showed that while response inhibition has been shown to be impaired in human volunteers after THC administration, neither disruption of endocannabinoid signaling nor administration of a CB1R agonist had clear observable behavioral effects on stop-signal task performance (66). Differential effects on adolescent mice have been shown by pharmacological inhibition of the fatty acid amide hydrolase (FAAH), the major enzyme implicated in anandamide degradation. Murine models showed that it prevented cognitive disruptions induced by distracting cues in adolescent mice. In particular, these protective effects were indicated by increased accuracy and correct responses and decreased premature responses selectively in the distractor trials (67).

## Cannabinoids in Neuropsychiatric Disorders Characterized by Impulsivity and Response Inhibition Impairments

In the last decade, a number of studies investigated the use of CBD in neuropsychiatric disorders characterized by motor and cognitive impulsivity/compulsivity, such as Attention Deficit/Hyperactivity Disorder (ADHD) and Tourette syndrome. In regard to ADHD, it is known that two regions of the endocannabinoid system, the hippocampus and cerebellar vermis, have been identified as being uniquely influenced by an interaction between cannabis use and the altered brain circuitry of ADHD diagnosed individuals and in a recent study (68). ADHD participants had impaired response inhibition combined with less fronto-parietal/striatal activity, regardless of cannabis use history and cannabis use did not impact behavioral response inhibition. Also, cannabis use was associated with hippocampal and cerebellar activation, areas rich in cannabinoid receptors, in control group but not ADHD participants (ibidem). Also, a childhood diagnosis of ADHD, but not cannabis use in adulthood, was associated with executive dysfunction. Earlier initiation of cannabis use may be linked to poor cognitive outcomes and a significantly greater proportion of the ADHD group began using cannabis before age 16. Regular cannabis use starting after age 16 may not be sufficient to aggravate longstanding cognitive deficits characteristic of ADHD (69).

In Tourette syndrome, Δ9-THC efficaciously reverses peripheral but not central motor tics. Δ9-THC may reduce ambulatory movements and evoke premonitory urges in some pediatric patients. The small “therapeutic window” in juveniles suggests that CBD may not effectively treat motor tics in children and may even exacerbate tics in a population of patients with Tourette syndrome (70). However, a recent systematic review suggests that there is insufficient evidence to provide guidance on the use of cannabinoids for mental health conditions within a regulatory framework, since only a single, small RCT for ADHD compared pharmaceutical THC:CBD with placebo and no significant effect was seen on the primary outcome, ADHD symptoms (71). Also, two small studies demonstrated no significant benefit of pharmaceutical THC:CBD compared to placebo on Tic/Tourette symptoms (ibidem).

## Cannabidiol as a Candidate Treatment for Addictions and Disorders of Motivation

Cannabidiol (CBD) is one such drug that shows therapeutic potential in a broad range of neurological and psychiatric diseases. Emerging preclinical and clinical evidence also indicates that CBD regulates different aversive and appetitive memory processes (10, 72). In preclinical studies in humans and animals, CBD reduces drug-motivated behavior, attenuates withdrawal effects, and limits cravings. Consistent with results demonstrating antagonizing effects of CBD on THC-induced pharmacological actions, cannabis containing higher vs. lower levels of CBD decreases the incentive salience of cannabis-related stimuli in smokers, and a case study reported a reduction in cannabis withdrawal symptoms following CBD administration (73). In contrast to its effects on opioid-motivated behaviors, CBD has less apparent influence on psychostimulant reward and reinforcement (ibidem). The endocannabinoid system might be of relevance to impulsivity and decision-making. Administration of high doses of CB1R agonists increases impulsive behaviors, whereas the administration of low doses of CB1 antagonists improves set-shifting performance and reduces the number of impulsive responses (74). In a rat model of gambling disorder, the administration of a CB1/2 agonist improved choice performance in a suboptimal group of rats, as evaluated using the rat gambling task (rGT). Although it is premature to propose that the stimulation of CB1/2R may provide a treatment for gambling individuals prone to poor decision-making the study from Gueye and colleagues implicates the cannabinoid system in the processing of cost-benefit decision-making. It should be noted that, up to date cannabidiol (or cannabidiol/THC mixtures) have mainly been studied in substance use disorders: CBD and THC mixtures showed positive effects in reducing short-term withdrawal and craving in cannabis use disorders, while studies on schizophrenia and comorbid substance use are lacking (75). Currently, there are only clinical studies on substance use disorder, while the effects of cannabidiol in other types of addiction or disorders of motivation have not been studied in randomized clinical trials yet.

## Conclusions

The inclusion of GD in the “substance related and addictive disorders” chapter of DSM-5 recognizes the disorder as a prototypical behavioral addiction, characterized by symptom clusters of loss of control, craving/withdrawal, and neglect of other areas of life.

The adoption of a RDoC approach facilitates the identification of the neurobiological factors underlying the disorder by breaking up a complex psychiatric disorder into its components and domains and identifying the corresponding constructs and subconstructs, thus rendering the process more tangible and experimentally addressable. Importantly, RDoC constructs relate to biological and behavioral measures and may also help in identifying endophenotypes for the disorder. Therefore, recent research in GD is focusing on the identification of the neurobiological underpinnings of most employed behavioral tasks related to decision making and response inhibition (e.g., Iowa Gambling Task, Delayed Discounting Task, and Stop Signal Task), to identify the neural correlates of the disorder's symptomatologic clusters and domains.

These deficits are associated with the RDoC domains of Positive Valence Systems (and its constructs of Approach motivation and Reward learning) and Cognitive Control (mainly its construct Response inhibition), respectively. Consistent with the RDoC matrix, deficits in preference-based decision-making have been identified in GD with the utilization of the IGT, revealing an involvement of numerous brain areas such as the striatum, amygdala, and OFC. Evidence regarding aberrant reward learning mechanisms are less robust, nevertheless they were hypothesized to be related with reward-based cognitive inflexibility and associated with an involvement of the OFC and ventral striatum, as highlighted in the RDoC matrix. Lastly, deficits in Cognitive control and particularly in the subconstruct of response inhibition have been identified in the disorder, using the SST and the Go/No-Go task, revealing the involvement of a fronto-striatal circuit and of the pre-supplementary motor area (pre-SMA). Further research is needed to expand our knowledge regarding the constructs of the disorder and how they correlate with the clinical presentation of the disorder as well as with the abnormalities at a neurocircuits level of explanation. The endocannabinoid system has been shown to play a crucial role in the regulation of different aversive and appetitive memory processes related to addiction mechanisms. In a RDoC perspective, EC role has been highlighted in Positive Valence Systems (reward attainment) and Cognitive Systems (declarative memory and working memory) has been highlighted. A putative role of endocannabinoids in response inhibition mechanisms has also been hypothesized, deriving evidence from the use of CBD during the Go/No-Go task. Nevertheless, evidence is still scarce to clearly determine which disorders may benefit from CBD administration based on impaired RDoC domains and constructs. Some insights derive from studies conducted on neuropsychiatric disorders characterized by motor and cognitive impulsivity and deficits in executive functions and response inhibition (e.g., ADHD, Tourette syndrome). This might also lead to hypothesize an involvement of EC system in the new sensorimotor domain of RDoC. Animal and human neuroimaging studies have also shown differential effects of THC vs. CBD on specific tasks and regional brain metabolism and, especially, in specific sub-populations. This might reflect the case of other compounds and substances, such as caffeine, whose effects clearly depends on the age window of administration. What is crucial to consider in this context is the developmental trajectory of the disorder: studies in this field have already unraveled the this dimension for response flexibility—an executive function that resembles simple motor inhibition in that both depend on sustained attention and the inhibition of prepotent responses, that differs from motor inhibition in that only the former requires subjects to execute an alternative response when the appropriate cue appears—in bipolar disorder (76). The evidence of differences in cognitive control between children and adults has also been highlighted by fMRI studies showing that children are more susceptible to interference and in prefrontal function and improvements in cognitive less able to inhibit inappropriate responses than adults. Effective interference suppression in children was associated with prefrontal activation in the opposite hemisphere relative to adults. In contrast, effective response inhibition in children was associated with activation of posterior, but not prefrontal, regions activated by adults. Children failed to activate a region in right ventrolateral prefrontal cortex that was recruited for both types of cognitive control by adults. Thus, children exhibited immature prefrontal activation that varied according to the type of cognitive control required (77). These differences may account for the differential choice of a specific compound that may exert an effect on the trajectory of development of brain networks and neurotransmettitorial signaling only in certain age groups.

More recently in the field of behavioral addictions, other contributions have disentangled similar mechanisms of faulty inhibitory control and faulty decision-making with preference for immediate reward to long-term gains in subjects with Internet gaming disorder (IGD) (78). Specifically, IGD subjects performing the Go/No Go task in fMRI showed greater impulsivity and lower activity of the right supplementary motor area/presupplementary motor area and showed increased activation in orbito-frontal cortex in gain trials and decreased anterior cingulate cortex activation in loss trials implicating enhanced reward sensitivity and decreased loss sensitivity (ibidem). Furthermore, regular or chronic IGD resulted in reduced brain's dopamine indicated by lower dopamine transporter density and lower dopamine D2 receptor occupancy in the brains of videogame players. In summary, further research is needed to elucidate the potential mechanisms involved in the regulation of response inhibition and reward-related decision-making that may be partially or fully mediated by EC system in behavioral addictions and, more specifically, in GD.

## Data Availability Statement

The original contributions presented in the study are included in the article/supplementary material, further inquiries can be directed to the corresponding author/s.

## Author Contributions

SP, AM, and NM have contributed to the ideation, drafting, and preparation of the paper. All authors contributed to the article and approved the submitted version.

## Funding

Research reported in this publication was supported by the National Institute on Drug Abuse of the National Institutes of Health under Award Number (R21DA042271).

## Conflict of Interest

The authors declare that the research was conducted in the absence of any commercial or financial relationships that could be construed as a potential conflict of interest.

## Publisher's Note

All claims expressed in this article are solely those of the authors and do not necessarily represent those of their affiliated organizations, or those of the publisher, the editors and the reviewers. Any product that may be evaluated in this article, or claim that may be made by its manufacturer, is not guaranteed or endorsed by the publisher.
